# Involvement of NADH Oxidase in Biofilm Formation in *Streptococcus sanguinis*

**DOI:** 10.1371/journal.pone.0151142

**Published:** 2016-03-07

**Authors:** Xiuchun Ge, Xiaoli Shi, Limei Shi, Jinlin Liu, Victoria Stone, Fanxiang Kong, Todd Kitten, Ping Xu

**Affiliations:** 1 Philips Institute for Oral Health Research, Virginia Commonwealth University, Richmond, VA 23298, United States of America; 2 State Key Laboratory of Lake Science and Environment, Nanjing Institute of Geography and Limnology, Chinese Academy of Science, Nanjing 21008, China; 3 Department of Microbiology and Immunology, Virginia Commonwealth University, Richmond, VA 23298, United States of America; University of Florida, UNITED STATES

## Abstract

Biofilms play important roles in microbial communities and are related to infectious diseases. Here, we report direct evidence that a bacterial *nox* gene encoding NADH oxidase is involved in biofilm formation. A dramatic reduction in biofilm formation was observed in a *Streptococcus sanguinis nox* mutant under anaerobic conditions without any decrease in growth. The membrane fluidity of the mutant bacterial cells was found to be decreased and the fatty acid composition altered, with increased palmitic acid and decreased stearic acid and vaccenic acid. Extracellular DNA of the mutant was reduced in abundance and bacterial competence was suppressed. Gene expression analysis in the mutant identified two genes with altered expression, *gtfP* and *Idh*, which were found to be related to biofilm formation through examination of their deletion mutants. NADH oxidase-related metabolic pathways were analyzed, further clarifying the function of this enzyme in biofilm formation.

## Introduction

Biofilms are ubiquitous in nature, in that nearly every microbial species has mechanisms for adhering to surfaces and other cells in mixed-species communities. According to the National Institutes of Health (NIH), biofilms could be responsible for over 80% of microbial infections [1]. In the United States alone, biofilm-related infections account for an estimated 1.7 million infections and 99,000 associated deaths each year (Centers for Disease Control and Prevention Report, 2007). Biofilms are known to help protect bacteria from abiotic stresses, antimicrobials and assaults of the host immune system. Within biofilms, bacterial cells are encased in an extracellular matrix that is typically composed of exopolysaccharides (EPS), proteins, lipids and nucleic acids [2]. Biofilm matrix is closely associated with adhesion of bacteria to biotic and abiotic surfaces and with cohesion of cells in the biofilm [2, 3]. Numerous studies have focused on various signals and mechanisms that control expression of the genes required for matrix production and biofilm formation. Quorum-sensing signals such as acyl-homoserine lactone, *Pseudomonas* quinolone signal and autoinducer-2 have been reported to play important roles in biofilm formation in bacteria [4–6].

*Streptococcus sanguinis* is a normal inhabitant of the oral cavity and one of the pioneer colonizers of tooth surfaces. Biofilm formation is important for *S*. *sanguinis* to colonize and interact with other bacterial inhabitants or pathogens in mixed-species communities in the oral cavity. In our previous study, we identified several genes involved in biofilm formation in this bacterium [7].

During glycolysis, bacterial cells produce NADH from NAD^+^. NADH oxidase has been recognized as playing an important role in maintaining glycolysis by producing NAD^+^ from NADH and, thus, maintaining NAD^+^/NADH balance. In *Streptococcus agalactiae*, inactivation of the *nox* gene encoding NADH oxidase was shown to reduce growth under aerobic conditions [8], while growth was not affected by *nox* inactivation in *Streptococcus pneumoniae* under aerobic or anaerobic conditions [9]. In *Streptococcus mutans*, Nox is responsible for the majority of NADH-dependent oxygen consumption and is involved in adaption to acidic and oxidative stresses [10]. In *S*. *pneumoniae* and *S*. *agalactiae*, *nox* inactivation attenuates virulence in animal models [8, 9]. In addition, the efficiency of competence for genetic transformation was significantly altered in an *S*. *pneumoniae nox* mutant [9]. Therefore, the *nox* gene plays important roles in many biological functions in streptococci.

In *Streptococcus gordonii*, the expression of the *nox* gene was increased in biofilms relative to planktonic cells [11]. Mutation of the *rex* gene, encoding a NADH- and NAD^+^-sensing transcriptional regulator, caused biofilms to decrease and to exhibit a more porous and rugged architecture in *S*. *mutans*[12]. Rex was shown to interact with the *nox* gene in a regulatory loop [13]. These results suggest that the *nox* gene may be involved in biofilm formation in streptococci; however, direct evidence is lacking.

Over the course of screening a library of *S*. *sanguinis* mutants for biofilm deficiency, we identified the *S*. *sanguinis nox* gene (SSA_1127) as being involved in biofilm formation. The *nox* gene was annotated as encoding an H_2_O-forming NADH dehydrogenase in *S*. *sanguinis*. In another study, we report that the a recombinant Nox protein (rNox) of *S*. *sanguinis* has the activity of an H_2_O-forming NADH oxidase under aerobic conditions (Ge et al., submitted for publication), which is consistent with NADH oxidases in *S*. *pneumoniae* [9], *S*. *mutans* [14] and *S*. *agalactiae* [8]. In this study, we confirmed a role for the *nox* gene in biofilm formation under anaerobic conditions and examined possible mechanisms by which the *nox* gene could influence biofilm formation. We found that rNox possesses NADH dehydrogenase activity under anaerobic conditions and that the *nox* mutant exhibited a dramatic reduction in biofilm formation. The *nox* mutant also exhibited alterations in fatty acid composition and decreases in membrane fluidity, extracellular DNA (eDNA) and bacterial competence. The *gtfP* and *Idh* genes, which were significantly down-regulated in the *nox* mutant, were shown to be involved in biofilm formation.

## Materials and Methods

### Ethics Statement

The study was conducted in accordance with the Declaration of Helsinki and saliva was collected using a protocol approved by the Virginia Commonwealth University Institutional Review Board (protocol HM10244).

### Saliva collection

Subjects at least 21 years of age, not on medication or ill, and who had not eaten or drunk anything other than water, or brushed their teeth within 60 min prior to collection were recruited. Saliva collection and processing were performed as described previously [15, 16]. Briefly, subjects chewed on paraffin to stimulate saliva production and then expectorated into a 50-ml centrifuge tube for 5 minutes. The tube was capped and placed on ice. After collection, saliva was mixed at 4°C for 20 minutes with 2.5 mM dithiothreitol to prevent protein aggregation and then centrifuged at 5000 x *g* for 20 min. The supernatant was transferred to a new tube, mixed with 3 volume of sterile dH_2_O, and filter sterilized. Samples from 6–10 subjects were pooled and stored at -20°C until use.

### Bacterial strains, growth and antibiotics

*S*. *sanguinis* strain SK36 and its mutants (Table 1) were grown in brain heart infusion (BHI) broth or agar (BD, San Jose, CA) at 37°C under anaerobic conditions (10% CO_2_, 10% H_2_ and 80% N_2_ with a catalyst) as described previously [17]. Biofilm medium containing 1% (w/v) sucrose (BM) was used for biofilm formation [7]. Bacto Todd Hewitt broth (BD, San Jose, CA) supplemented with 2.5% (v/v) horse serum (Fisher scientific, Pittsburgh, PA) (TH-HS) was used for transformation. Antibiotics including 500 μg/ml kanamycin, 10 μg/ml erythromycin (Fisher scientific, Pittsburgh, PA) and 100 μg/ml spectinomycin (Sigma-Aldrich, St. Louis, MO) were used for mutant construction and culture.

**Table 1:** Strains and primers in this study.

Strain or primer	Description*	Source or application
***S*. *sanguinis***		
SK36	Human plaque isolate	Kilian et al. (1989)
*∆nox*	Kan^r^; *ΔSSA_1127*:: *aphA-3*	This study
*∆nox_compl*	Erm^r^; *SSA_1127*:: *erm*	This study
*∆ldh*	Kan^r^; *ΔSSA_1221*:: *aphA-3*	Xu et al. (2011)
*∆gtfP*	Kan^r^; *ΔSSA_0613*:: *aphA-3*	Xu et al. (2011)
JFP36	Erm^r^; *ΔSSA_0169*:: *pSerm*	Turner et al. (2009)
**Primers**		
nox_F1	CCATCTACCGACTTGTCTGAAAC	*nox* upstream
nox_R1	GCCATTTATTCCTCCTAGTTAGTCAACTCATAAGAATAGTCCTACCTTA	*nox* upstream
Kan_F2	TGACTAACTAGGAGGAATAAATGGCTAAAATGAGAATAT	*aphA-3*
Kan_R2	CATTATTCCCTCCAGGTACTAAAACAATTCATCCAGT	*aphA-3*
nox_F3	GTTTTAGTACCTGGAGGGAATAATGATTACTCAAGCAGCTTTGAAAGC	*nox* downstream
nox_R3	GTAGGAAATAACCAATCGGAAGAAT	*nox* downstream
nox_compl_F1	nox_F1	*nox* upstream&ORF
nox_compl_R1	TGTAATCACTCCTTCTCACTATTTATTTTGCTTTCAAAGCTGCTTGA	*nox* upstream&ORF
Erm_F2	TAAATAGTGAGAAGGAGTGATTACATGAACAA	*erm*
Erm_R2	TTATTTCCTCCCGTTAAATAATAG	*erm*
nox_compl_F3	CTATTATTTAACGGGAGGAAATAAGAAAATGAGTCTGGGATAAATTTCCA	*nox* downstream
nox_compl_R3	nox_R3	*nox* downstream
nox_rp_F	GACGACGACAAGATCAGTAAAATCGTTGTAGTTGGTGCAA	Cloning of *nox* ORF
nox_rp_R	GAGGAGAAGCCCGGTTATTTTGCTTTCAAAGCTGCTTGA	Cloning of *nox* ORF
gtfP_L	GCCCAAATTCTCAACCGTTAC	qRT-PCR of *gtfP*
gtfP_R	ATCTTGCCCTTGACTTGGTAG	qRT-PCR of *gtfP*
ldh_L	ATGCTCGTTCTGTCCATGCCTACA	qRT-PCR of *ldh*
ldh_R	TGCTCCAATTTGACACCTGCAACG	qRT-PCR of *ldh*
gyrA_L	AGCTGATTGCCTTGATTGCAGAC	qRT-PCR of *gyrA*
gyrA_R	ATCCGCAAATTTACGCTTGACCT	qRT-PCR of *gyrA*

* Kan, kanamycin; Erm, erythromycin.

### Deletion and complementation of the *nox* gene

The *nox* open reading frame (ORF) in *S*. *sanguinis* SK36 was replaced by a promoterless kanamycin cassette (*aphA-3*) as described previously [17]. Briefly, three pairs of primers nox_F1 and nox_R1, nox_F3 and nox_R3, and kan_F2 and kan_R2 (Table 1) were used for PCR-amplification of 1-kb upstream and downstream flanking regions of the *nox* gene, and promoterless *aphA-3*, respectively. The three PCR-amplified fragments were combined by second-round PCR amplification using primers nox_F1 and nox_R3. The final linear recombinant PCR amplicon was transformed into *S*. *sanguinis* SK36 to obtain the *nox*-deleted mutant using kanamycin for selection. The mutant was confirmed by colony-PCR amplification using nox_F1 and nox_R3, followed by amplicon sequencing.

The *nox* mutant was complemented by a similar strategy. Upstream (1-kb) plus ORF of the *nox* gene, promoterless erythromycin cassette (*erm*) and 1-kb downstream of the *nox* gene were PCR amplified and then combined to obtain a recombinant PCR amplicon in which the *nox* ORF was followed by the *erm* cassette. The recombined amplicon was transformed into the *nox* mutant to obtain a complemented strain of the *nox* mutant using erythromycin for selection. The strain was confirmed as for the *nox* mutant. The primers used are listed in Table 1.

### Biofilm formation analysis

Biofilm formation of *S*. *sanguinis* wild-type and mutants was examined in 96-well or 12-well microtiter plates (Greiner Bio-One, Monroe, North Carolina). Overnight BHI-cultured strains were diluted 100-fold in BM on plates and incubated 16 h at 37°C under anaerobic conditions for biofilm formation [7]. After measuring absorbance at 450 nm for bacterial growth, the plate wells were gently washed with deionized water (dH_2_O), and stained with 50 μl of 0.4% (w/v) crystal violet (Fisher scientific, Pittsburgh, PA) for 15 min at room temperature. After 3 times washing with dH_2_O, the biofilm stain was dissolved in 200 μl of 33% (v/v) acetic acid and then 100 μl transferred for measuring absorbance at 600 nm. For confocal laser scanning microscopy, *S*. *sanguinis* biofilm formed on 12-well plates was stained with 10 μM SYTO 9 (Life Technologies, Grand Island, NY) for 15 min at room temperature. After washing, the biofilm was observed with a Leica TCS-SP2 AOBS confocal laser scanning microscope (VCU core facilities) using a laser wavelength of 488 nm and emission wavelengths of 495–525 nm [18]. A series of green fluorescent x-y sections in the z plane of the biofilm was scanned and obtained. Images were analyzed with Image J (National Institutes of Health) [18]. For biofilm formation on human protein-coated plates, 96-well plates were coated with 100 μg/ml fibrinogen, 10 μg/ml fibronectin, 10 μg/ml collagen type I or 10 μg/ml laminin (Sigma-Aldrich, St. Louis, MO) overnight at 4°C, respectively [19]. After washing, the coated plates were used for biofilm formation assays as above. Biofilm formation was performed in human saliva via pre-coating plate wells with human saliva or mixing saliva with BM [20]. For the pre-coating experiment, 100 μl of 25% filter-sterilized saliva was added to the plate wells and incubated at 37°C with gentle shaking. After 2 h incubation, the plate wells were washed with sterile PBS and used for biofilm formation analysis as above. For the experiment with saliva-mixed medium, saliva was added to BM medium to a final concentration of 10% (v/v) prior to analysis as above. PBS was used as a control for saliva.

### Expression and purification of rNox protein

The cloning, expression and purification of recombinant *S*. *sanguinis* Nox protein in *E*. *coli* was performed as described previously [21]. Briefly, the *S*. *sanguinis nox* gene was PCR-amplified using primers nox_rp_F and nox_rp_R (Table 1), cloned into pET-46 Ek/LIC vector (Novagen, Madison, WI) and expressed in *E*. *coli* BL21(DE3)pLysS (Novagen, Madison, WI) according to the manufacturer’s protocols. The expressed rNox protein with N-terminal His tag was isolated using BugBuster buffer (Novagen, Madison, WI) and purified using a His•Bind® Column Chromatography kit (Novagen, Madison, WI) as described in the manufacturer's protocols.

### NADH dehydrogenase activity assay

To identify alternative electron receptors for the rNox in the absence of oxygen, the oxidation of NADH to NAD^+^ by the rNox was analyzed under anaerobic conditions. Reagent preparation and the assays by NanoDrop® ND 1000 Spectrophotometer (Thermo scientific, Wilmington, DE) were performed in an anaerobic chamber. Each reaction mixture contained 50 mM potassium phosphate buffer (pH 7.0), 1 mM β-NADH, 1 mM each of the potential electron receptors and the purified rNox [22]. The reduction of the acceptors by rNox was monitored at the following wavelengths: FAD, 450 nm; dichlorophenolindophenol (DCIP), 600 nm; menadione, 340 nm; K_3_(FeCN_3_)_6_, 420 nm; cytochrome c, 550 nm; CoQ_10_, 340 nm; and XTT, 480 nm [22]. Protein concentrations were determined by the Bradford method [23] using bovine serum albumin as a standard. All chemicals were purchased from Sigma-Aldrich (St. Louis, MO).

### Membrane fluidity

Membrane fluidity was quantified by fluorescence anisotropy of 1,6-diphenyl 1,3,5-hexatriene (DPH; Sigma-Aldrich, St. Louis, MO) as described previously[24]. Briefly, exponential-growth-phase *S*. *sanguinis* planktonic cells cultured anaerobically in BM medium were harvested, washed twice with PBS (pH 7.4) and then incubated with 5 μM DPH at 37°C for 1 h. Unlabeled cells were used as a scattering reference. The fluorescence polarization was measured at 37°C using a Cary Eclipse Fluorescence spectrophotometer (VCU Department of Chemistry Instrumentation Facility) with excitation at 360 nm and emission at 430 nm. Fluorescence anisotropy was calculated by the formula A = (I_II_—I_⊥_)/(I_II_ + 2I_⊥_), where I_II_ and I_⊥_ were the fluorescence intensities parallel and perpendicular to the direction of excitation light, respectively.

### Determination of bacterial fatty acid composition

The bacterial cell fatty acid composition was determined by GC/MS as described [25]. *S*. *sanguinis* cells were harvested from overnight cultures in BM medium under anaerobic conditions by centrifugation and washed twice with dH_2_O. The cell pellets were mixed with 0.5 ml 1 N sodium methoxide for 1 min and the fatty acid methyl esters were extracted by addition of 0.3 ml hexane containing methyl-10-undecenoate (Sigma-Aldrich, St. Louis, MO) as an internal standard. GC/MS analysis was carried out on Varian Saturn GC/MS (VCU core facilities) equipped with a Restek Stabilwax-DA column (30 m × 0.25 mm × 0.5 μm). Carried gas (H_2_) velocity was 1 ml/min. Injection and detection temperature were 230 °C and 260°C, respectively. The oven temperature was increased from 100°C to 240°C at 5°C/min and maintained for 20 min. The peaks with their retention times were identified by MS. Each fatty acid composition was expressed as percentage of the total content of fatty acids.

### eDNA assay

*S*. *sanguinis* cultures incubated overnight in BM medium anaerobically were centrifuged at 6,000 rpm for 10 min at 4°C and the supernatants were collected and passed through 0.45 μm filters (EMD Millipore, Billerica, MA) to eliminate residual cells. Half milliliter of the supernatant was mixed with 0.5 ml TE buffer saturated with phenol-chloroform-isoamyl alcohol (25:24:1; Sigma-Aldrich, St. Louis, MO) [26]. After vortexing for 30 s, the mixture was centrifuged at 16,000 ×g for 5 min at 4°C. The aqueous phase (0.4 ml) was transferred into a new tube and mixed with 40 μl 3M sodium acetate (pH5.2) and 1 ml 100% ethanol. After placement at -20°C for 10 min, the mixture was centrifuged at 16,000 ×g for 10 min at 4°C and the pellets were collected. After air drying, the pellet was suspended in 100 μl dH_2_O and then DNA concentration was determined by measuring absorbance at 260 nm using a NanoDrop® ND 1000 Spectrophotometer.

### Competence assay

Competence of *S*. *sanguinis* strains was determined by transformation with pJFP96, a suicide plasmid containing the spectinomycin resistance gene (*aad*9) with ~1 kb upstream and downstream of the SSA_0169 [27]. Briefly, overnight cultures of *S*. *sanguinis* strains were diluted 200-fold into pre-warmed TH-HS and incubated microaerobically for 2–5 hrs. Aliquots of 330 μl culture at various incubation times were transferred into pre-warmed microfuge tubes containing 70 ng *S*. *sanguinis* competence-stimulating peptide (CSP) and 50 ng pJFP96, and incubated at 37°C for 1 h. Cells were serially diluted, plated on BHI agar plates with and without spectinomycin and grown microaerobically at 37°C for 2 d. The efficiency of transformation was defined as the ratio of spectinomycin-resistant colonies to total CFU.

### Microarray analysis

*S*. *sanguinis* SK36, *nox* mutant and complemented strain cells cultured anaerobically in BM medium were harvested from the exponential growth phase and used for microarray analysis [17]. RNA from the sample cells was isolated through lysozyme lysis, mechanical disruption with FastPrep® lysing matrix B and purified using RNeasy mini kit (Qiagen, Valencia, CA) [17]. DNA was removed by treatment in columns with DNase I during purification. The microarray was performed on spotted microarray slides obtained from the Pathogen Functional Genomics Resource Center at JCVI according to the manufacturer’s protocol [17]. Cy3 and Cy5 were used for labeling cDNA of SK36 and the *nox* mutant/complemented strain, respectively. The microarray data were obtained from scanning of hybridization slides using a GenePix 4100A microarray scanner (Molecular Devices, Downingtown, PA), and analyzed using Spotfinder and Midas programs (TM4 software suite) to obtain the expression ratio of *nox* mutant/complementation to the wild-type. The microarray data have been deposited in the NCBI Gene Expression Omnibus (GEO) under the accession number of GSE68492.

### qRT-PCR

*S*. *sanguinis* RNA from the microarray assay was used to perform real-time reverse transcription PCR (qRT-PCR) for detecting the expression change of genes *ldh* and *gtfP* in the *nox* mutant. For all reactions, first-strand cDNA was synthesized in a 20 μl reaction mixture containing 4 μl of 5× first-strand buffer, 100 ng RNA, 1.5 μg random primers, 1 μl of 10 mM dNTP mix, 1 μl of 0.1 M DTT, 1 μl of RNaseOUT™ Recombinant RNase Inhibitor (40 U/μl) and 1 μl of SuperScript™ III reverse transcriptase (200 U/μl) following the manufacturer’s protocol (Life Technologies, Grand Island, NY). The qRT-PCR reaction was composed of 5 μl 2× SYBR Green PCR master mix (Life Technologies, Grand Island, NY), 10 pmol of each paired primer, 1 μl of 50-fold diluted cDNA template and dH_2_O up to 10 μl using Applied Biosystems 7500 Fast Real-Time PCR system (Life Technologies, Grand Island, NY). The housekeeping gene *gyrA* was used as a normalization control.

### Statistical analysis

All data were obtained in at least triplicates. For data on confocal microscopy and qRT-PCR, one sample t-test was applied to analyze the values of mutant or complemented mutant that differ from 1. The electron receptor data were statistically analyzed by ANOVA followed by Tukey’s HSD test. The microarray data were analyzed by t-test with correction of false discovery rate (FDR). Other data were statistically analyzed by t-test. The significance was set as P-value <0.05.

## Results

### The *nox* gene is involved in biofilm formation

**Fig 1:**
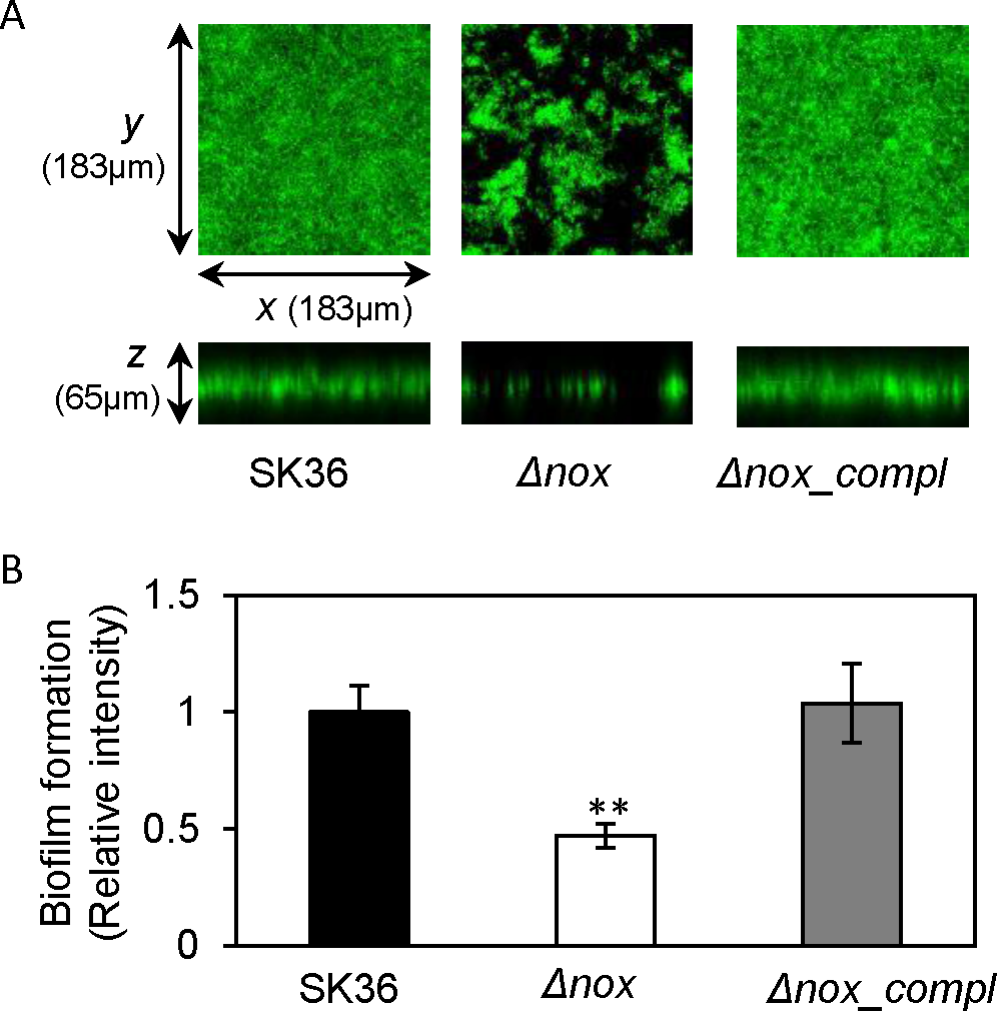
Reduction in biofilm formation in the *nox* mutant as assessed by confocal laser scanning microscopy. A, biofilm formation on a polystyrene surface detected using a confocal laser scanning microscope. B, quantification of biofilm formation in A. Δ*nox*, the *nox* mutant; *Δnox_compl*, the complemented strain of the *nox* mutant. **, significant difference with P < 0.01 between SK36 and *nox* mutant. Data obtained at least in triplicates.

We first examined anaerobic growth of overnight cultures of a *nox* mutant, a complemented *nox* mutant, and the wild-type parent, SK36, in BHI broth by dilution plating. There were no significant differences among the three strains (data not shown). We next examined biofilm formation of the *nox* mutant in BM medium because it has been shown to support abundant biofilm formation by *S*. *sanguinis* SK36 cells [7]. Biofilms were formed on the polystyrene surfaces of microtiter plate wells overnight under anaerobic conditions and then examined using confocal laser scanning microscopy. The results showed that the amount of biofilm formed by the *nox* mutant was significantly less than the wild-type (P-value < 0.01) and had a very different structure, whereas biofilm amounts and structure were restored by complementation of the *nox* mutation (Fig 1).

**Fig 2:**
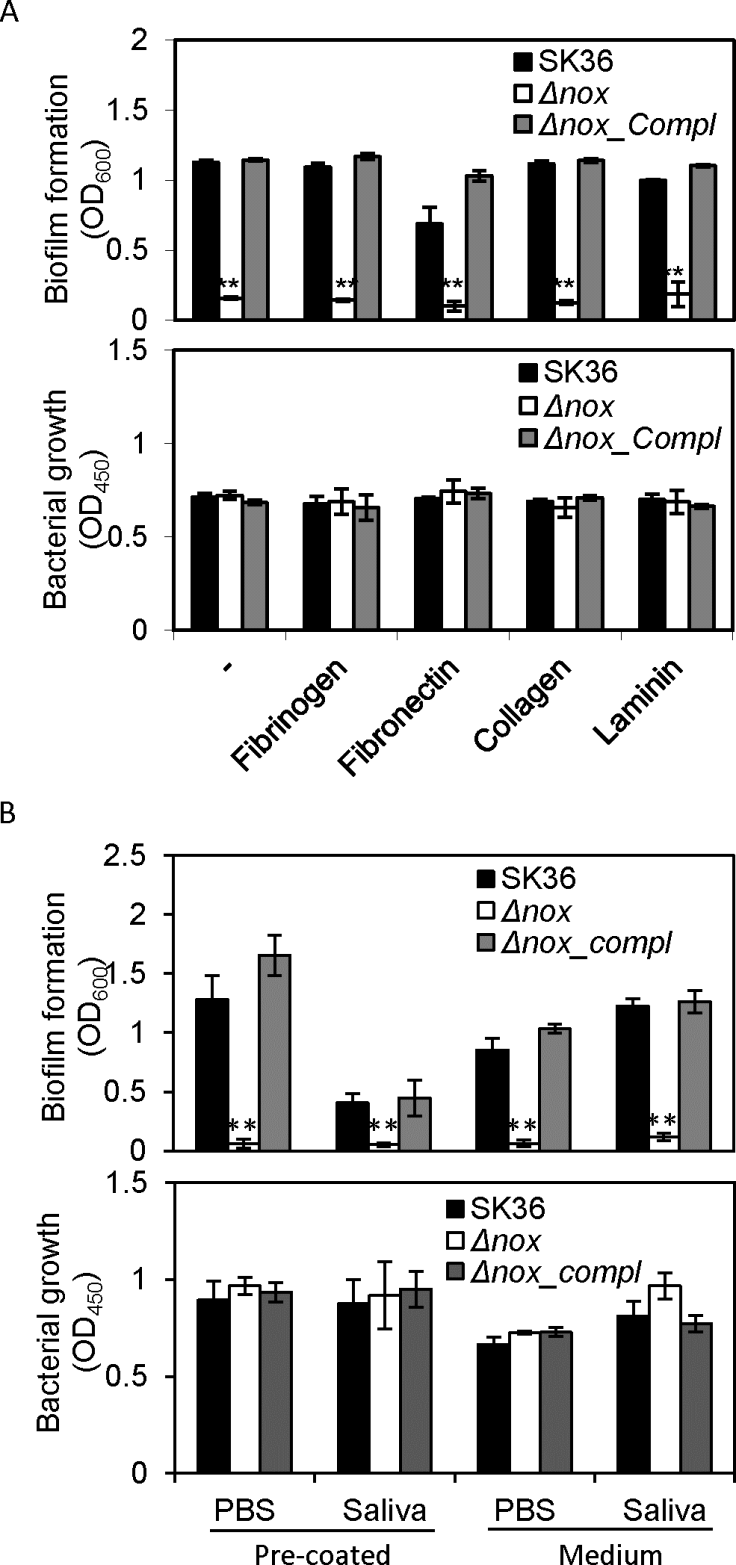
Decrease in biofilm formation of the *nox* mutant with human plasma, extracellular matrix proteins and saliva. A, biofilm formation (OD_600_) and growth (OD_450_) in BM on plate wells pre-coated with human plasma and extracellular matrix proteins. B, biofilm formation (OD_600_) in BM on plate wells pre-coated with human saliva (pre-coated) and in BM medium mixed with saliva (medium). (OD_450_), growth in BM (pre-coated) or BM mixed with saliva (medium). Δ*nox*, the *nox* mutant; *Δnox_compl*, the complemented strain of the *nox* mutant. **, significant difference with P < 0.01 compared to SK36. Data obtained at least in triplicates.

To examine whether the *nox* mutant formed reduced biofilm on host cell constituents, human plasma protein fibrinogen and extracellular matrix proteins including fibronectin, collagen and laminin [28–31] were coated on the plate surface prior to assaying biofilm formation. The results confirmed that the *nox* mutant exhibited a significant reduction in biofilm formation on different extracellular matrix constituent-coated surfaces compared to the wild-type (Fig 2A). Complementation of the *nox* mutation restored biofilm formation to the same level as the wild-type. Further, the biofilm formation of the wild-type, mutant and complemented mutant in human saliva were examined. Both in wells pre-coated with saliva or BM medium mixed with saliva, biofilm formation was also dramatically decreased in the *nox* mutant compared to the wild-type (Fig 2B). There were no significant growth differences among the experimental strains in either medium, suggesting that changes in growth were not responsible for the reduced biofilm formation of the mutant (Fig 2). These results confirmed that the *nox* gene is involved in biofilm formation in *S*. *sanguinis*.

### NADH dehydrogenase activity of rNox in the absence of oxygen

**Fig 3:**
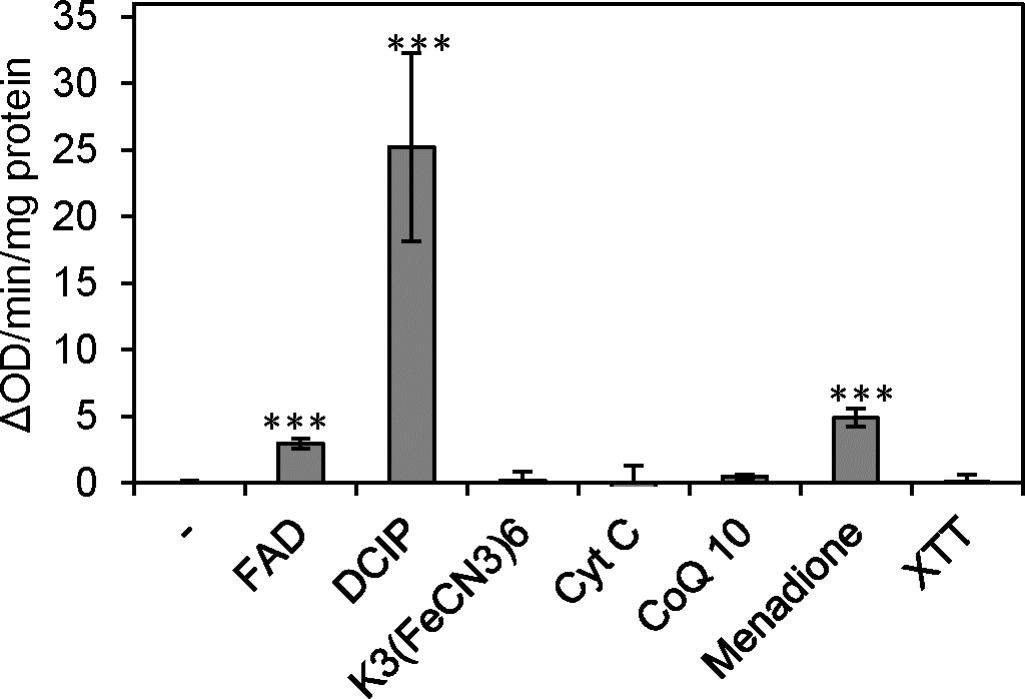
NADH dehydrogenase activity of the rNox protein. Reduction of electron receptors by the rNox measured by spectrophotometrically monitoring the following wavelengths: FAD, 450 nm; DCIP, 600 nm; menadione, 340 nm; K_3_(FeCN_3_)_6_, 420 nm; cytochrome c, 550 nm; CoQ_10_, 340 nm; XTT, 480 nm. ***, P < 0.001. Data obtained at least in triplicates.

Under aerobic conditions, oxygen is the electron acceptor that allows the *nox*-encoded NADH oxidase to oxidize NADH to NAD^+^ in *S*. *sanguinis* (Ge et al., submitted for publication). Here, other potential electron acceptors to allow the rNox protein to oxidize NADH to NAD^+^ were analyzed under anaerobic conditions. As shown in Fig 3, rNox could reduce DCIP, menadione and FAD in the presence of NADH, but not K_3_(FeCN_3_)_6_, cytochrome C, CoQ_10_ or XTT. This result suggests that there are electron acceptors besides oxygen that could serve as a substrate for the rNox protein. This could explain how mutation of the *nox* gene affected biofilm formation, which was assayed under anaerobic conditions (See Methods.)

### Membrane fluidity decrease in the *nox* mutant

**Fig 4:**
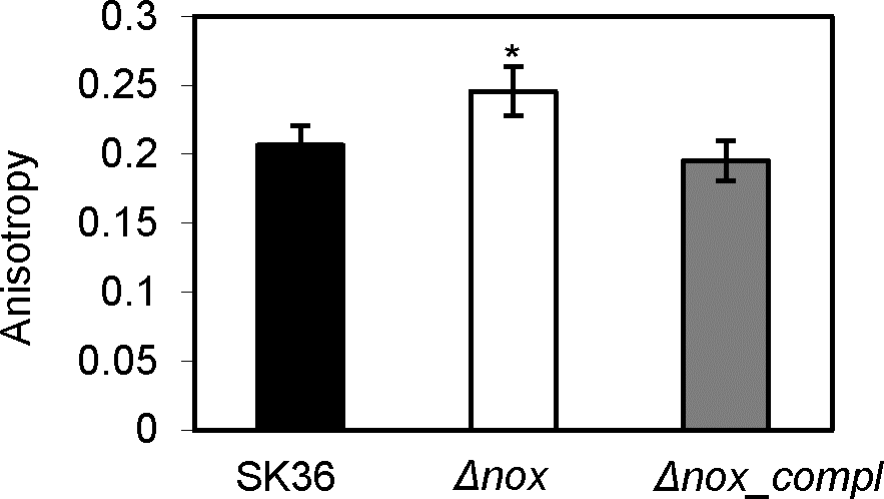
Change in membrane fluidity in the *nox* mutant. Δ*nox*, the *nox* mutant; *Δnox_compl*, the complemented strain of the *nox* mutant. *, significant difference with P < 0.05 compared to SK36. Data obtained at least in triplicates.

To further understand how the *nox* gene affects biofilm formation, we examined NADH oxidase-related metabolic pathways. We considered that the *nox* mutant might have an altered cell membrane, because NADH oxidase has been associated with fatty acid biosynthesis in *S*. *agalactiae* and *S*. *mutans* [8, 10]. The membrane fluidity in the *nox* mutant and the wild-type was assessed using a DPH anisotropy approach. In this assay, an increase in DPH anisotropy represents a decrease in membrane fluidity, and vice versa [24]; small changes in DPH anisotropy may reflect marked changes in membrane microviscosity [32]. In the *nox* mutant, the DPH anisotropy was significantly greater than the wild-type (Fig 4), indicating reduced membrane fluidity.

### Fatty acid composition change in the *nox* mutant

Membrane fluidity has been found to be linked with fatty acid composition [24]. The finding of membrane fluidity reduction implied that the levels of individual membrane lipids might change in the *nox* mutant. Thus, we ascertained the composition of fatty acids in the *nox* mutant and compared to the wild type. As shown in Table 2, the results indicated palmitic acid (C16:0) content was significantly increased but the levels of stearic acid (C18:0) and *cis*-vaccenic acid (C18:1, ω7) were significantly decreased in the *nox* mutant compared to the wild-type. These results suggested that deletion of the *nox* gene led to increase in the content of C16 fatty acids and to decrease in the content of C18 fatty acids in the membrane of *S*. *sanguinis* cells. The decrease in unsaturated fatty acid (*cis*-vaccenic acid) would be expected to result in decreased membrane fluidity [33], in agreement with our findings (Fig 4).

**Table 2:** Comparison of fatty acid composition in the *nox* mutant and wild-type.

Fatty acids	SK36	Δ*nox*	p-value	Δ*nox*_compl	p-value
	(Mean ± SD)	(Mean ± SD)		(Mean ± SD)	
C12:0	2.9 ± 0.33	3.5 ± 2.11	0.6641	2.6 ± 0.83	0.5201
C14:0	22.6 ± 2.41	23.5 ± 2.29	0.6814	18.5 ± 4.79	0.2555
C14:1	1.6 ± 1.04	4.1 ± 3.54	0.3014	2.6 ± 0.27	0.1857
C16:0	18.1 ± 2.20	27.5 ± 3.79	0.0209	20.7 ± 4.70	0.4406
C16:1	12.5 ± 3.75	12.7 ± 3.15	0.9399	10.6 ± 2.56	0.5083
C18:0	15.8 ± 3.33	5.4 ± 1.55	0.0080	10.9 ± 1.81	0.0885
C18:1, ω9	6.1 ± 3.07	7.4 ± 4.56	0.7014	10.4 ± 3.44	0.1852
C18:1, ω7	15.2 ± 2.30	5.7 ± 1.64	0.0044	16.1 ± 10.98	0.8943
C19:0, cy(ω9,10)	3.4 ± 1.60	6.1 ± 0.74	0.0533	3.8 ± 2.64	0.8247
Unidentified fatty acid	1.8 ± 0.94	4 ± 1.60	0.1063	3.9 ± 2.22	0.2040

### Diminishment of eDNA in the *nox* mutant

**Fig 5:**
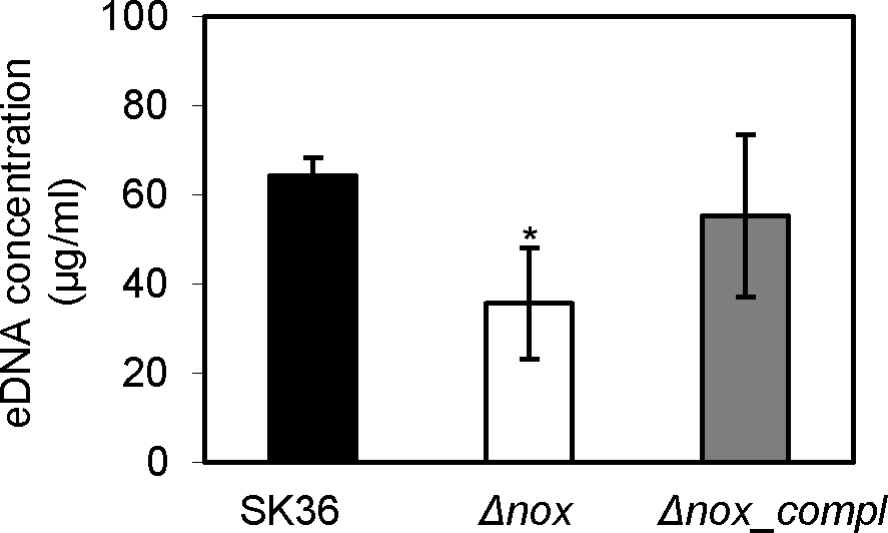
Change in eDNA concentration in the *nox* mutant. Δ*nox*, the *nox* mutant; *Δnox_compl*, the complemented strain of the *nox* mutant. *, significant difference with P < 0.05 compared to SK36. Data obtained at least in triplicates.

DNA release has been demonstrated to play an important role in biofilm formation and is associated with bacterial competence [34–37]. Thus, levels of eDNA in the *nox* mutant were examined and compared to the wild-type. As shown in Fig 5, the eDNA concentration in the *nox* mutant was significantly less than that in the wild-type.

### Competence suppression in the *nox* mutant

**Fig 6:**
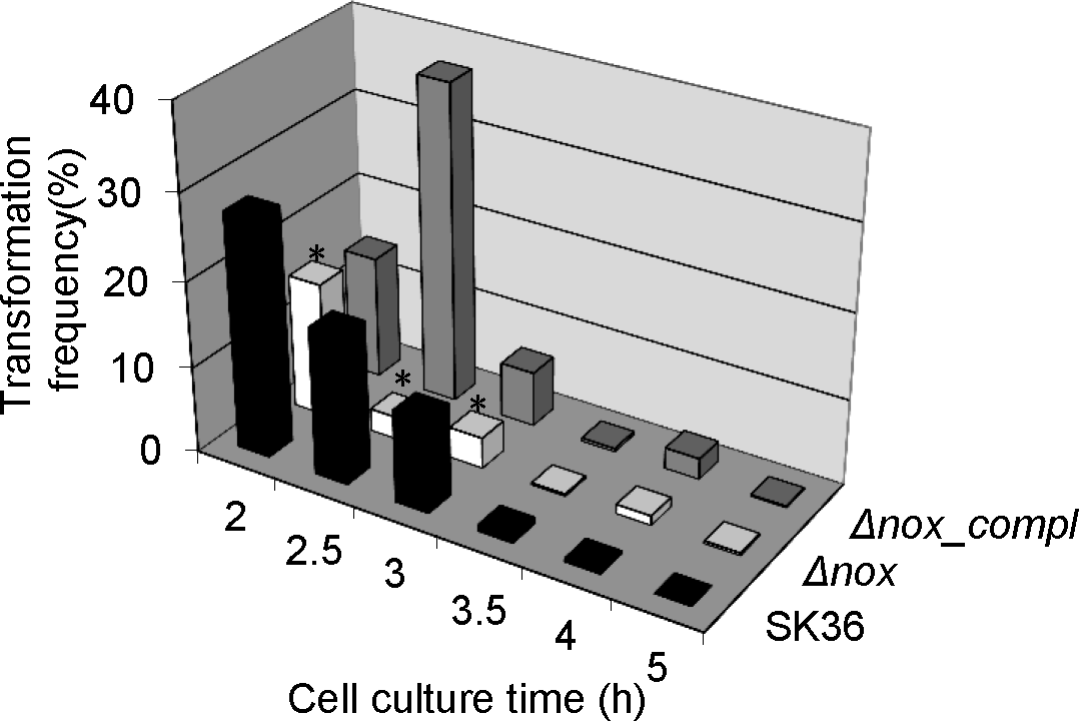
Change in competence in the *nox* mutant. Bacterial cells were incubated 2–5 hrs prior to addition of DNA. Δ*nox*, the *nox* mutant; *Δnox_compl*, the complemented strain of the *nox* mutant. *, significant difference with P < 0.05 compared to SK36. Data obtained at least in triplicates.

We also compared the competence of the *nox* mutant and the wild-type by transformation with pJFP96 [27], a plasmid that inserts a spectinomycin resistance gene into the chromosome at a dispensable location by allelic exchange [38]. As shown in Fig 6, transformation with pJFP96 was significantly suppressed in the *nox* mutant compared to wild-type, even though CSP was added. This result differs from that seen in *S*. *pneumoniae*, where mutation of the *nox* gene lowered transformation efficiency only when exogenous CSP was not added [9]. It’s not clear why the timing of competence development was slightly altered in the complemented mutant, but the important outcome is that the *nox* mutant was significantly less competent than SK36 at the first three time points, and longer incubation did not increase its competence.

### Nox-controlled biofilm-related genes

To reveal whether *nox* deletion possibly influenced the expression of other genes involved in biofilm formation, global gene expression of the *nox* mutant and its complement were compared to the wild-type in BM medium using two-color spotted microarrays. All three strains were grown under anaerobic conditions. The microarray data showed that 13 genes were significantly down-regulated and 71 genes up-regulated in the *nox* mutant, and most were partially or fully restored in the complemented mutant strain (S1 Table). From the metabolic pathway analysis, three of the down-regulated genes, *ldh* and *acoA* and *acoB* (components of acetoin dehydrogenase Acdh), are responsible for converting pyruvate into lactate and acetyl-CoA in pyruvate metabolism. Amongst the up-regulated genes, there were three, *ald*, *ackA* and *adhE*, that participate in pyruvate metabolism. In addition, several operons associated with sucrose and galactose metabolism were up-regulated in the *nox* mutant. These include *gal* (SSA_1003 to SSA_1010) containing *gtfA* (SSA_1006) and *lac* operons (SSA_1692 to SSA_1699) for galactose metabolism from sucrose. It was interesting that genes SSA_0509 to SSA_0531, containing an operon for ethanolamine metabolism, showed increased expression. This operon was acquired by horizontal gene transfer [39]. NADH oxidase has been demonstrated to function as an important NADH-oxidizing system in glycolysis in *Streptococcus agalactiae*[8]. Therefore, these changes in gene expression may be a response to the *nox* deficiency to ensure normal glycolysis in *S*. *sanguinis*. (See below.)

**Fig 7:**
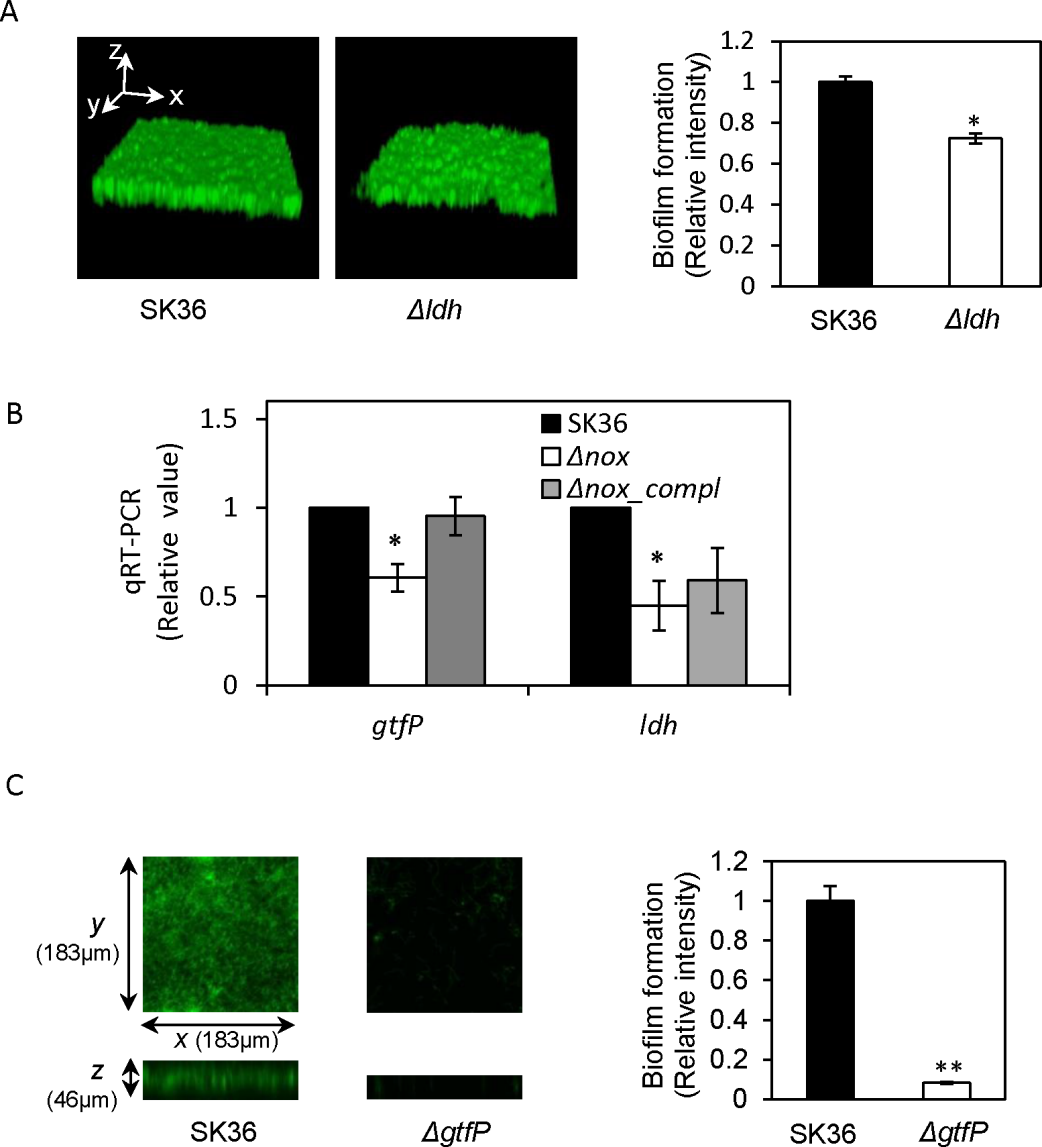
Biofilm and gene expressions changes of the *nox-*related mutants. A, biofilm structural comparison of the *ldh* mutant (left) and SK36 by confocal laser scanning microscopy. B, qRT-PCR for the expressions of *ldh* and *gtfP* genes in the *nox* mutant using *gyrA* as an internal control. C, biofilm decrease of the *gtfP* mutant (left). Δ*ldh*, the *ldh* mutant; Δ*nox*, the *nox* mutant; *Δnox_compl*, the complemented strain of the *nox* mutant; Δ*gtfP*, *gtfP* mutant; P-value significant difference in triplicates compared to SK36 *, P < 0.05; **, P < 0.01.

Using our comprehensive single-gene deletion library [17], we picked mutants deleted for each of the 13 down-regulated genes and examined their biofilm formation. The vast majority of these mutants did not display significant changes in biofilm formation (data not shown) except that the *ldh* mutant exhibited an uneven biofilm surface and lower biofilm abundance than the wild-type (Fig 7A). The significant decrease in expression of *ldh* in the *nox* mutant was also verified by qRT-PCR analysis (Fig 7B).

The *gtf* genes have been implicated in biofilm formation in *Streptococcus mutans* [40, 41]. These include *gtfB*, *gtfC and gtfD*. *S*. *sanguinis* possesses only a single *gtf* gene, *gtfP*. We examined *gtfP* expression in the microarray assay of the *nox* mutant but were unable to draw a conclusion because its expression levels were too low in most of the experiments. We further quantified its expression using qRT-PCR. The qRT-PCR data indicated *gtfP* expression was detectable and significantly lower in the *nox* mutant than in the wild-type (Fig 7B). We then examined the biofilm formation of a *gtfP* deletion mutant and found that it exhibited a 12-fold reduction compared to the wild-type (Fig 7C). These results suggest that reduced expression of *ldh* and *gtfP* may be involved in the biofilm reduction of the *nox* mutant.

## Discussion

Nox orthologs have been demonstrated to possess NADH oxidase activity in *S*. *pneumoniae* [9], *S*. *mutans* [14] and *S*. *agalactiae* [8]. We have also verified that *S*. *sanguinis* Nox does indeed function as an H_2_O-forming NADH oxidase (Ge et al., submitted for publication). Without oxygen, however, other electron receptors, such as DCIP, menadione and FAD [14] (Fig 3), could be reduced by the Nox protein in the presence of NADH. Although we do not know the exact electron acceptor(s) employed by the Nox in *S*. *sanguinis* cells, FAD is a possible candidate because *S*. *sanguinis* has the metabolic pathway for FAD synthesis and BM medium contains riboflavin. Regardless, to our knowledge, this is the first time a streptococcal NADH oxidase has been shown to possess NADH dehydrogenase activity under anaerobic conditions. Remarkably, we identified a marked deficiency in biofilm formation, changes in membrane fluidity and fatty acid composition, release of DNA, and changes in gene expression in the *nox* mutant under anaerobic conditions. Thus, Nox is important for cell physiology even in the absence of oxygen.

Using confocal laser scanning microscopy, biofilm formation was observed to significantly decrease in the *S*. *sanguinis nox* mutant (Fig 1). Similar findings were also shown on plate surfaces coated with human extracellular matrix or plasma proteins as well as human saliva (Fig 2). These results indicate that the *nox* gene is involved in biofilm formation in *S*. *sanguinis* not only on an abiotic surface but also on biotic surfaces.

Under aerobic conditions, *nox* inactivation was demonstrated to impair cell growth in *S*. *agalactiae* [8]. The same study also showed that deficiency in aerobic growth of the *nox* mutant was mostly caused by an underlying defect in fatty acid biosynthesis due to reduction in total fatty acid yield, and suggested that NAD^+^ depletion in a *nox* mutant probably affects acetyl-CoA production, a precursor for fatty acid biosynthesis. In our study, biofilm formation was defective in the *nox* mutant of *S*. *sanguinis*, but bacterial growth was not influenced by deletion of *nox* under anaerobic conditions (Fig 2A). Furthermore, fatty acid abundance was not significantly changed in the *nox* mutant compared to the wild type in the same culture conditions (data not shown).

Microarray data indicated that *adhE*, *ackA*, and genes required for ethanolamine metabolism were significantly elevated in their expression in the *nox* mutant (S1 Table). The *adhE* gene encodes a bifunctional enzyme that acts as an acetaldehyde dehydrogenase, which converts acetyl-CoA into acetaldehyde with concomitant oxidation of NADH to NAD^+^, as well as an alcohol dehydrogenase that converts acetaldehyde to ethanol. The *ackA* gene encodes acetate kinase, which produces ATP from acetyl phosphate. Ethanolamine metabolism has been proposed to contribute to pathogenesis in bacteria such as *Salmonella enterica* subsp. *enterica* serovar Typhimurium and *E*. *coli* [42]. Ethanolamine catabolism occurs in an ethanolamine-specific microcompartment, where acetaldehyde derived from ethanolamine can be catabolized to ethanol by EutG (which encodes an alcohol dehydrogenase), or to acetyl-CoA by EutE (which encodes an acetaldehyde dehydrogenase) and then converted into acetyl phosphate by EutD (which encodes a phosphotransacetylase). Acetyl phosphate can diffuse freely into the cytoplasm and generates ATP through AckA. Within the microcompartment, CoA and NAD^+^/NADH can be recycled by EutD, EutE and EutG. In the bacterial cytoplasm, Pta plays the same role as EutD, converting acetyl-CoA into acetyl phosphate[42].

**Fig 8:**
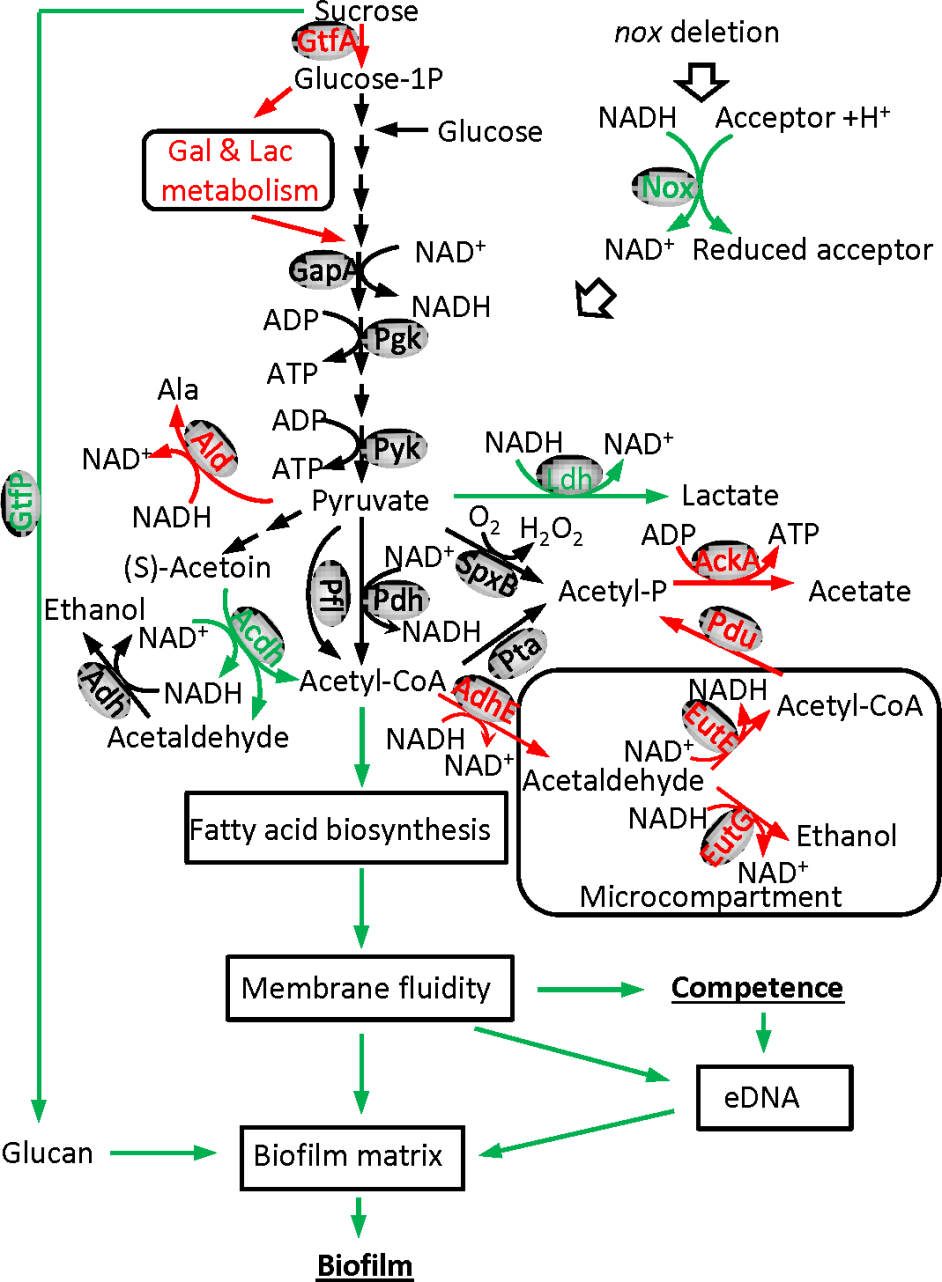
Deduced mechanisms of the *nox* gene in relation to biofilm formation in *S*. *sanguinis*. Deletion of *nox* leads to cessation of the *nox*–mediated oxidation of NADH to NAD^+^. This causes decreased expression (green) of *ldh* and two genes encoding components of acetoin dehydrogenase, *acoA* and *acoB*, and increased expression (red) of *adhE*, *ackA*, *ald* and ethanolamine utilization genes including *eutG*, *eutE* and *pdu*. These gene expression changes affect the associated pathways to partially compensate for the imbalance of NAD^+^/NADH caused by *nox* deletion. Meanwhile, *gtfA* (red) expression increases and *gtfP* (green) expression decreases, which may cause more sucrose influx into energy metabolism rather than exopolysaccharide biosynthesis (e.g. glucan). Increased consumption of acetyl-CoA by AdhE may influence fatty acid biosynthesis to alter the membrane fatty acid components and to subsequently decrease the membrane fluidity and may suppress the release of eDNA. The inhibition of DNA release and glucan biosynthesis impairs biofilm matrix and results in defective biofilm formation. Green arrow, reduced reaction or function in the *nox* mutant; black arrow, normal reaction or function; red arrow, increased reaction or function; hollow arrow, function lost as a result of the *nox* deletion.

In *S*. *sanguinis*, the orthologs of *eutE* (SSA_0523) and *eutG* (SSA_0514) are present in the ethanolamine operon but *eutD* is missing. However, another phosphotransacetylase encoded by *pduL* (SSA_0527), which has been demonstrated in *S*. *enterica* subsp. *enterica* serovar Typhimurium [43], is present and located within the ethanolamine operon in *S*. *sanguinis*. These three genes showed increased expression in the *nox* mutant (S1 Table). In addition, the expression of the *pta* gene (SSA_1207) did not change in the microarray analysis of the *nox* mutant (data not shown), suggesting cytoplasmic phosphotransacetylase encoded by *pta* might not be involved in the metabolism above. These results suggest that deletion of *S*. *sanguinis nox* may cause AdhE to oxidize NADH into NAD^+^, concomitantly converting acetyl-CoA into acetaldehyde which may enter the ethanolamine specific microcompartment to be catabolized into ethanol by EutG and acetyl phosphate by EutE and PduL, with concomitant recycling of CoA and NAD^+^/NADH in the microcompartment, and acetyl phosphate could then diffuse into the cytoplasm and generate ATP via AckA. In addition, the *ald* gene, involved in metabolizing pyruvate into alanine with concomitant oxidation of NADH to NAD^+^, was up-regulated in the *nox* mutant, which could enhance oxidation of NADH to NAD^+^. The down-regulation of two *acdh* genes *acoA* and *acoB*, involved in reduction of NAD^+^ into NADH, could conserve NAD^+^ in the *nox* mutant. (See Fig 8) Therefore, it is possible that the elimination of Nox-mediated conversion of NADH to NAD^+^ in *S*. *sanguinis* cells due to *nox* deletion may be offset by decreases in *acoA* and *acoB* and increases in *ald*, *adhE*, *ackA* and ethanolamine catabolism. These compensatory changes may lead to diminished levels of acetyl-CoA, which though sufficient for normal cell growth, may affect downstream metabolism such as fatty acid biosynthesis (see below).

The composition of fatty acids was shown to be altered in the *S*. *sanguinis nox* mutant, as reflected by decreases in stearic acid and *cis*-vaccenic acid and an increase in palmitic acid (Table 2). Obviously, these results indicated that *nox* deletion affected the composition of fatty acids in *S*. *sanguinis* despite the unchanged total amount of fatty acids. These changes may be due to the limited level of acetyl-CoA in the *nox* mutant (see above). Moreover, the membrane fluidity of the *nox* mutant was decreased compared to the wild-type (Fig 4). Fourier transform infrared spectrometry has demonstrated that changes in membrane fluidity are directly associated with the level of unsaturated fatty acids in bacterial biological membranes [33]. Thus, the decrease in membrane fluidity in the *nox* mutant may result from the reduction in the contents of unsaturated fatty acids, such as *cis*-vaccenic acid. In *Pseudomonas aeruginosa*, lipid analysis showed a significant decrease in the uneven-numbered chain phospholipids and a slight increase in long chain phosphatidylethanolamine in biofilm compared to planktonic cells [44]. The results revealed that a decrease in membrane fluidity and lipid stability in the bilayer may be required for biofilm formation in *P*. *aeruginosa*. In *S*. *mutans*, anthraquinones could suppress biofilm formation on hydroxyapatite and the anthraquinone-treated *S*. *mutans* cells showed a significant decrease in membrane fluidity. Based on these reports, our results suggest the change in composition of fatty acids and subsequent decrease in membrane fluidity may be one of the causes of the biofilm defects observed in the *nox* mutant. Membrane fluidity is critical for maintaining the properties of the bacterial membrane and the functions of membrane-associated proteins, such as permeability of the lipid bilayer, protein mobility, protein–protein interactions and active transport processes [33, 43, 45–47]. Alterations in the membrane permeability and the functions of membrane-associated proteins due to decrease in membrane fluidity may affect the transport/translocation or assembly of eDNA (see below) and extracellular biofilm matrix in the *S*. *sanguinis nox* mutant.

Bacterial eDNA has been identified to play an important role in biofilm formation as well as dispersal [37]. The crucial role of eDNA in stabilizing bacterial biofilm matrix has been revealed in both Gram-negative and Gram-positive bacteria such as *P*. *aeruginosa*, *Staphylococcus aureus* and *S*. *gordonii* [36, 48, 49]. Additionally, eDNA has been shown to promote dispersal in biofilm by inhibiting settling of motile progeny cells in *Caulobacter crescentus* [50]. In our study, eDNA was significantly reduced in the *nox* mutant compared to the wild-type (Fig 5). This result suggests that eDNA decrease may be one of the reasons for the impaired biofilm formation seen in the *nox* mutant. In streptococci, biofilm biomass has been revealed to be decreased in competence-defective mutants [34, 51] and DNA release has been demonstrated to be associated with cell competence [34, 35]. Our results showed that transformation frequency was significantly decreased in the *nox* mutant (Fig 6), suggesting competence was attenuated due to deletion of the *nox* gene. These results suggest that eDNA reduction in the *S*. *sanguinis nox* mutant may be also affected by a decrease in cell competence. In addition, the decrease in membrane fluidity may also affect the uptake of exogenous DNA to attenuate competence.

The *gtfB*, *gtfC* and *gtfD* genes have been reported to act as glucosyltransferases for exopolysaccharide biosynthesis by utilizing sucrose [52, 53]. Moreover, these three genes have been reported to be involved in biofilm formation in *S*. *mutans* [54]. In *S*. *sanguinis*, like many other streptococci, *gtfP* is the only ortholog of these three genes. Our results showed that deletion of *gtfP* caused almost complete loss of *S*. *sanguinis* biofilm formation (Fig 7C). Apart from *gtfB*, *gtfC* and *gtfD*, *S*. *mutans* has another *gtf* gene, *gtfA*[52–54]. The ortholog of *gtfA* gene is extensively present in other streptococci and encodes sucrose phosphorylase responsible for converting sucrose into D-fructose and alpha-D-glucose 1-phosphate. In *S*. *sanguinis*, *gtfA* gene is located within a *gal* operon for galactose metabolism and its mutant did not show a decrease in biofilm formation (data not shown). It is interesting that the expression of *gtfP* was decreased (Fig 7B) but the expression of *gal* and *lac* operons containing *gtfA* was increased in the *S*. *sanguinis nox* mutant (S1 Table). These results indicate that the biofilm defect in the *nox* mutant may also be associated with reduction in *gtfP* expression. Meanwhile, an increase in *gtfA* expression and a decrease in *gtfP* expression in the *nox* mutant might cause more sucrose influx into energy metabolism rather than exopolysaccharide biosynthesis for biofilm formation. Furthermore, we also found deletion of *ldh* led to a more uneven biofilm surface and less biofilm formation than the wild-type (Fig 7A) and that expression of *ldh* was significantly decreased in the *nox* mutant (Fig 7B), possibly implicating *ldh* in the biofilm defect caused by the *nox* deletion. L-lactate dehydrogenase, encoded by *ldh*, converts pyruvate to L-lactate with concomitant oxidation of NADH to NAD^+^ to assure continued glycolysis in lactic acid bacteria including *Streptococcus* and *Lactobacillus* [55, 56]. It is possible that these functions of *ldh* may be replaced by ethanolamine catabolism in combination with *adhE* and *ackA* activity in the *S*. *sanguinis nox* mutant.

In conclusion, an *S*. *sanguinis nox* mutant exhibited a significant decrease in biofilm formation. The data suggest that the deficiency of biofilm formation by deletion of *nox* may be due to changes in metabolic pathways associated with NAD^+^/NADH balance and sucrose utilization, which might contribute to alteration in membrane fatty acid and subsequent decrease in membrane fluidity, suppression of DNA release, and reduction in the expression of biofilm-associated *gtfP* and *ldh* (Fig 8). Since the *nox* gene is widespread in other species of streptococci, continuation of this work may lead to a comprehensive elucidation of the underlying mechanisms of biofilm formation for streptococci.

## Supporting Information

S1 Table Genes significantly down-regulated and up-regulated in the *nox* mutant and restored in the *nox* complemented strain. (XLSX)
